# Splenectomy for wandering spleen with pedicle torsion in a 17-year-old: a case report

**DOI:** 10.3389/fmed.2025.1646831

**Published:** 2025-08-14

**Authors:** Guangchao Liu, Yifei Shen, Cheng Jiao, Yao Zhang, Xin Zhang, Qiujing Meng, Wei Liu

**Affiliations:** Department of General Surgery, Bethune International Peace Hospital, Shijiazhuang, Hebei, China

**Keywords:** wandering spleen, abdominal pain, splenic pedicle torsion, splenic infarction, splenectomy, anticoagulant therapy

## Abstract

**Background:**

Wandering spleen is a rare disorder characterized by splenic displacement into the abdominal or pelvic cavity, predisposing to torsion and infarction. Congenital factors (long pedicle, lax ligaments) or acquired conditions (splenomegaly) are the usual causes.

**Case presentation:**

A 17-year-old female presented with 5 days of escalating abdominal pain. Imaging (contrast-enhanced CT and ultrasound) revealed a pelvic spleen with 360° pedicle torsion and partial infarction. After multidisciplinary review, emergency laparotomy was performed and splenectomy was undertaken. Post-operatively, low-molecular-weight heparin followed by aspirin was prescribed to mitigate post-splenectomy thrombocytosis-related thrombosis. The patient was discharged on day 18 without complications and remained well at follow-up.

**Conclusion:**

Early recognition, prompt splenectomy, and individualized anticoagulation guided by hematology input are essential to prevent complications in acute splenic torsion.

## 1 Introduction

Wandering spleen is a rare clinical condition with an estimated incidence of 0.2%, accounting for only 0.25% of splenectomy cases. While it predominantly affects children under 10 years (particularly infants) and women aged 20–40 (female-to-male ratio 7:1) (1, 2). This condition poses unique diagnostic and management challenges, especially in patients with a history of abdominal surgery or other underlying comorbidities (3, 4). The etiology involves both congenital and acquired mechanisms: Congenital causes include embryonic dysplasia of the gastric dorsal mesentery, leading to malformation of splenic ligaments (e.g., gastro-splenic ligament), whereas acquired factors encompass abdominal trauma, pregnancy-related hormonal changes, or postsurgical anatomical alterations (5). These abnormalities result in an elongated splenic pedicle and loss of ligamentous fixation, allowing the spleen to migrate freely within the abdomen. The hypermobility predisposes to pedicle torsion, a surgical emergency that compromises vascular supply, culminating in splenic infarction, with a high mortality rate if misdiagnosed (6, 7).

This case contributes three points to the literature. Irst, it confirms that wandering spleen can present in healthy adolescents without prior surgery or pregnancy. Second, a pelvic ectopic spleen mimicked gynecological pain, and early contrast-enhanced computed tomography (CT) confirmed the diagnosis. Third, intraoperative findings of 360° torsion with established infarction necessitated splenectomy, illustrating the decision pathway when splenopexy is no longer viable.

## 2 Case description

A 17-year-old female was admitted to our hospital on August 21, 2024, because she had intermittent abdominal pain for 5 days, which got worse the day before admission. The pain was colicky, mostly around the belly button and in the lower left part of the abdomen. She also experienced nausea and two episodes of vomiting, each occurring approximately 2–3 h after a meal. The vomitus consisted of undigested food and gastric secretions, with no blood or bile noted. There was no history of gastro-esophageal reflux disease (GERD), or bloody/black stools. She had previously been treated conservatively at a local hospital for suspected wandering spleen, but her symptoms persisted despite conservative therapy. Given the patient’s young age and the potential reversibility of the condition, initial conservative management was chosen to avoid unnecessary surgical intervention and to allow for further diagnostic evaluation, including imaging studies (Table 1). The day before admission, her abdominal pain became more frequent and severe. She was subsequently admitted to our hospital for further evaluation and management.

**Table 1:** Timeline with relevant data.

Date	Event
2024-08-17	The patient presented with mild abdominal pain and was diagnosed with wandering spleen at a local hospital.
2024-08-21	The patient’s abdominal pain intensified and became more frequent. She was diagnosed with wandering spleen and splenic pedicle torsion at our hospital and was admitted for treatment.
2024-08-27	The patient underwent exploratory laparotomy. Intraoperatively, the spleen was found in the pelvic cavity with a 360° torsion of the splenic pedicle and extensive splenic infarction. Splenectomy was performed.
2024-09-01	The patient began ambulation. Oral enteric-coated aspirin was initiated for anticoagulation due to postoperative thrombocytosis.
2024-09-14	Ultrasound examinations of the hepatic portal vein and lower extremity deep veins showed no thrombosis. The patient was discharged.
2024-10-27	Two months postoperatively, the platelet count decreased to below 400 × 10^9^/L. Anticoagulation therapy with aspirin was discontinued. The patient remains in good condition.

**Figure 1:**
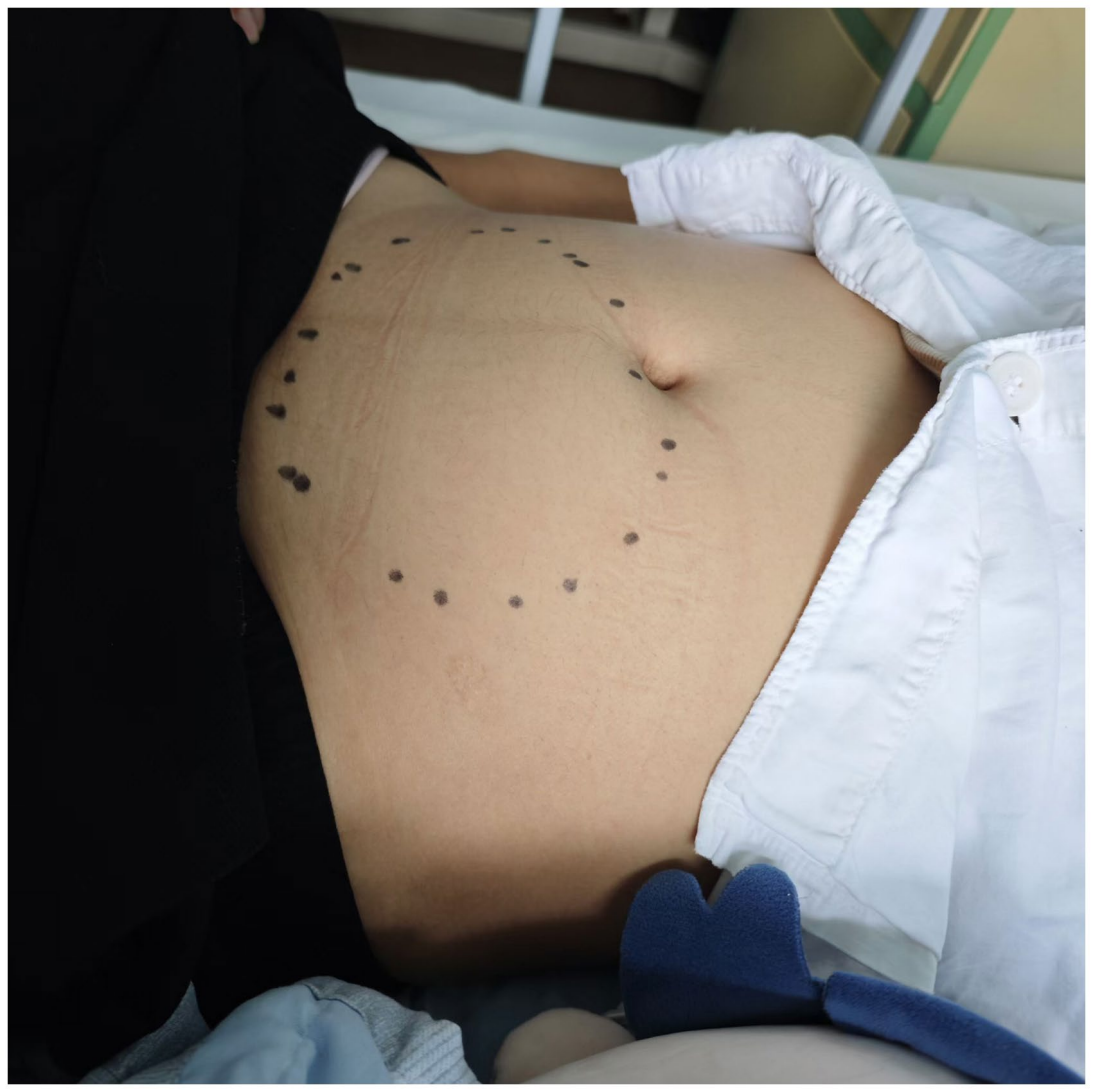
Abdominal examination reveals a palpable mass in the lower abdomen.

**Figure 2:**
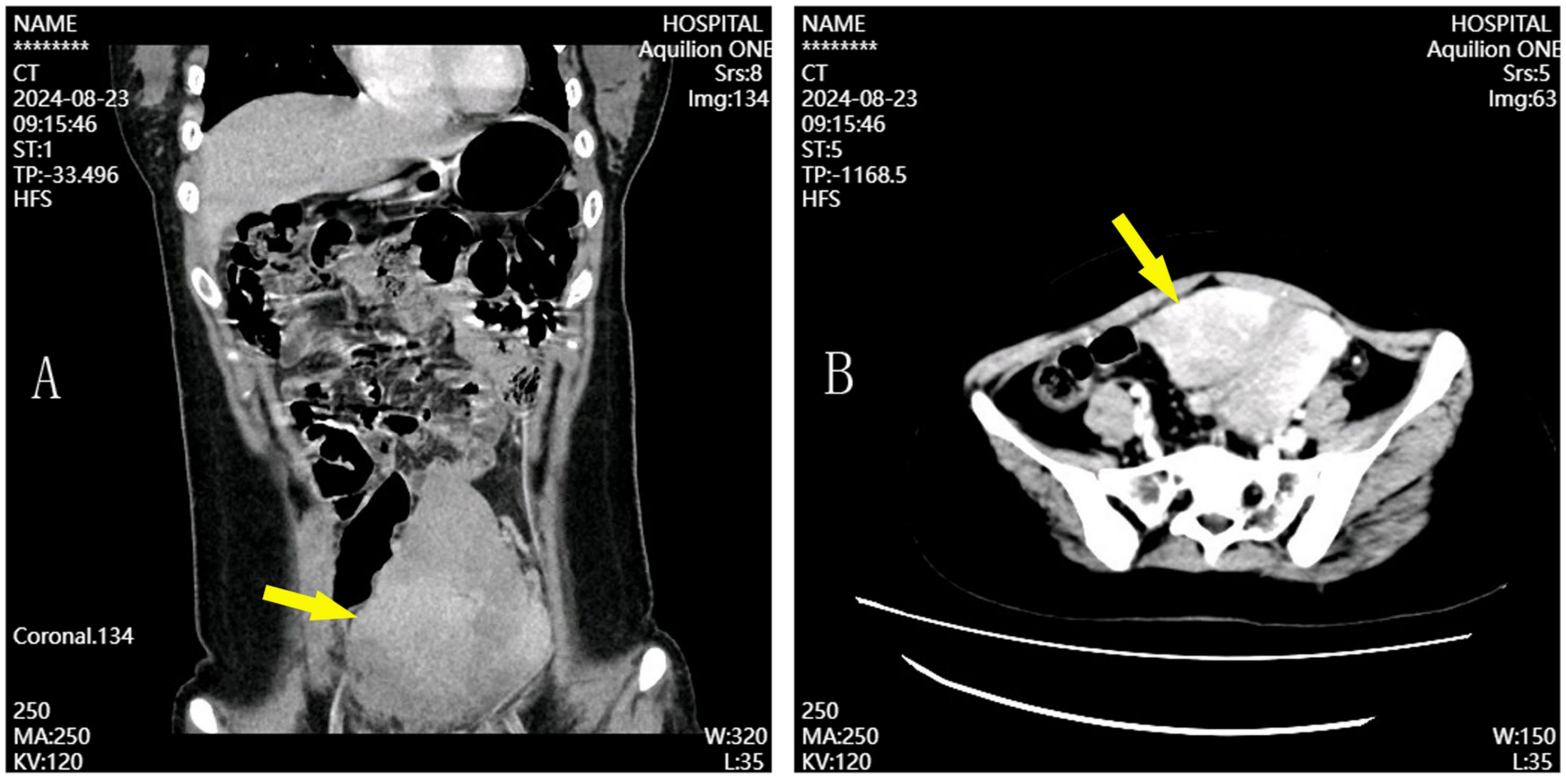
Coronal **(A)** and axial **(B)** CT scans showing splenic displacement and hypoperfusion consistent with infarction.

Upon admission, the patient’s body mass index (BMI) was recorded at 25.2 kg/m^2^. Cardiac and pulmonary examinations were unremarkable. Abdominal examination revealed slight distension of the lower abdomen, with deep tenderness noted around the umbilicus and the left side of the abdomen. No rebound tenderness was observed. A palpable mass, approximately 20 cm × 15 cm, was firm and moderately mobile in the lower and middle abdomen (Figure 1). Laboratory tests showed a white blood cell count of 5.63 × 10^9^/L, an absolute neutrophil count of 3.46 × 10^9^/L, hemoglobin of 111 g/L, a platelet count of 251 × 10^9^/L, and a C-reactive protein of 70.4 mg/L. Coagulation tests revealed a prothrombin time of 13.2 s, a fibrinogen level of 5.22 g/L, and a D-dimer level of 1.981 mg/L. Initial ultrasound examination revealed a large mass in the lower abdomen, consistent with a displaced spleen, exhibiting heterogeneous echotexture suggestive of possible areas of infarction. Subsequently, an enhanced abdominal CT scan was performed. During the arterial phase, the spleen was found in the pelvic cavity, significantly enlarged with uneven density reduction, and there were patchy, slightly dense shadows around the spleen. The splenic vein showed obvious rotation and tortuosity. In the venous phase, the infarcted area remained continuously without enhancement, and the surrounding normal splenic tissue exhibited decreased enhancement, likely due to congestion. A small amount of free fluid around the spleen suggested localized peritonitis (Figure 2). Acute appendicitis, ovarian torsion, and intestinal obstruction were among the differential diagnoses considered, but were excluded based on the specific imaging characteristics and clinical context. A multidisciplinary consultation involving radiologists, hematologists, and surgeons was convened. After thorough discussion and review of the clinical and radiological findings, the diagnosis of wandering spleen with splenic torsion and partial splenic infarction was confirmed upon admission.

**Figure 3:**
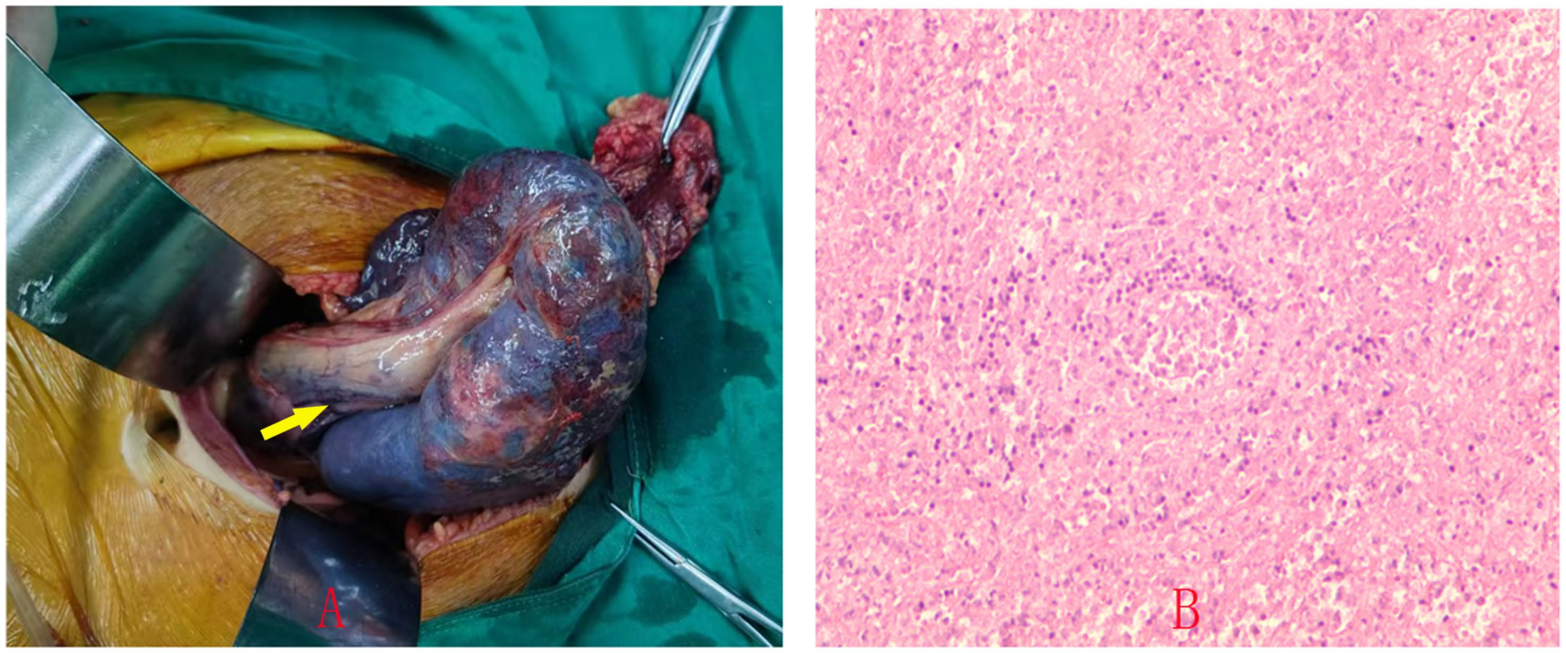
Intraoperative photo showing the spleen in the lower abdomen with absent perisplenic ligaments and partial infarction of the splenic parenchyma **(A)**. Postoperative pathology shows localized hemorrhage and necrosis within the splenic tissue **(B)**.

On August 27, 2024, the patient underwent a lower midline laparotomy. This approach was selected due to the large size of the spleen and the twisted splenic pedicle, which necessitated a thorough exploration of the abdominal cavity. Intraoperatively, the spleen was found in the lower abdomen and pelvic cavity, free and measuring approximately 18 cm × 10 cm. The spleen was dark red, with marked congestion and edema, hard texture, high surface tension, and mottled necrosis in some areas. The perisplenic ligaments were absent. The vessels at the splenic hilum were tortuous and congested, with the splenic artery and vein being particularly tortuous and rotated clockwise by approximately 360°. Some of the proximal small bowel mesentery was slightly twisted (Figure 3A). Intestinal adhesion lysis, splenectomy, and mesenteric repositioning were performed. A drain was placed in the pelvic cavity, exiting through the lower left abdomen and secured, to monitor for postoperative bleeding and infection. Postoperative pathology revealed localized hemorrhage and necrosis in the submitted splenic tissue (Figure 3B).

Postoperatively, the patient exhibited no signs of bleeding from the abdominal drain or incision. A hematologist was consulted to guide anticoagulation therapy and monitor coagulation parameters. Although vaccination against pneumococcus, meningococcus, and HiB is generally recommended post-splenectomy, these vaccines were not administered in this case due to resource limitations. However, the patient was given prophylactic antibiotic treatment to mitigate the risk of overwhelming postsplenectomy infection (OPSI). To prevent portal vein and lower extremity deep vein thrombosis, the patient received subcutaneous injections of 2,500 IU of low molecular weight heparin sodium once daily for anticoagulation. On postoperative day 2, laboratory tests revealed a platelet count of 197 × 10^9^/L, a prothrombin time of 13.6 s, and a D-dimer level of 0.779 mg/L. By postoperative day 3, the platelet count had risen to 490 × 10^9^/L, the prothrombin time had decreased to 9.1 s, and the D-dimer level had increased to 1.128 mg/L. On postoperative day 5, low molecular weight heparin sodium was discontinued, and oral aspirin enteric-coated tablets were initiated at 100 mg once daily for antiplatelet therapy. On postoperative day 7, the platelet count was 687 × 10^9^/L, the prothrombin time was 10.5 s, and the D-dimer level was 2.971 mg/L. By postoperative day 11, the platelet count had further increased to 693 × 10^9^/L, the prothrombin time had risen to 11.2 s, and the D-dimer level had decreased to 0.286 mg/L. On postoperative day 15, the platelet count was 675 × 10^9^/L. Abdominal and lower extremity deep vein color Doppler ultrasounds performed on postoperative days 7 and 14 showed no evidence of hepatic portal vein or lower limb vein thrombosis. The patient was discharged on postoperative day 18 and continued taking aspirin enteric-coated tablets at 100 mg once daily. Weekly blood counts during the first 2 months postoperatively showed platelet counts maintained between 400 × 10^9^/L and 600 × 10^9^/L. Anticoagulation therapy was discontinued when the platelet count fell below 400 × 10^9^/L at 2 months post-surgery. Follow-up abdominal and lower extremity deep vein ultrasounds after discharge revealed no thrombosis. The patient remained asymptomatic at 2-month follow-up with no evidence of thrombosis.

## 3 Discussion

Most patients with wandering spleen have no obvious symptoms before the torsion of the splenic pedicle. Even when a small range of torsion occurs and does not seriously affect the blood supply of the spleen, only mild abdominal discomfort symptoms appear (7). Some patients are diagnosed due to physical examinations or are incidentally discovered during other abdominal surgeries. A very small number of patients seek medical attention due to acute gastric retention or intestinal obstruction caused by the overly long splenic pedicle compressing the gastrointestinal tract (1, 8). The free spleen and vascular pedicle can also cause the displacement of the pancreas and the occurrence of acute pancreatitis (1, 8). Early diagnosis through physical examination and auxiliary tests like ultrasound and CT is crucial. Enhanced CT is especially important because it can show if the spleen is missing from the upper left abdomen and check how well blood is flowing to the spleen (9). When there are clear symptoms, it usually means the spleen has been damaged beyond repair, and splenectomy is the only surgical choice (10).

In this case, the patient presented with intermittent abdominal pain that intensified over 5 days, consistent with acute splenic torsion. A palpable abdominal mass and findings from the abdominal enhanced CT scan, including splenomegaly and partial infarction, were instrumental in confirming the diagnosis. These findings highlight the importance of considering wandering spleen in the differential diagnosis of unexplained abdominal pain, especially in young females, and underscore the value of early diagnostic imaging.

This case stands out compared to others in the literature. Wandering spleen usually affects children under 10 and women aged 20 to 40. Our 17-year-old patient is in the less common adolescent age group. Her symptoms, intermittent abdominal pain, nausea, and vomiting, resembled gastrointestinal issues, unlike typical acute abdominal pain. The spleen’s unusual pelvic location and a palpable abdominal mass added complexity to the initial diagnosis.

For asymptomatic patients with wandering spleen, splenopexy is a viable option if the spleen is of normal size and there is no evident thrombosis. This procedure, which involves fixing the spleen using autologous tissues or prosthetic aids, has proven effective with a low recurrence rate of splenic displacement (11). It not only preserves splenic function but also reduces postoperative hospital stays and associated costs. Moreover, it helps prevent life-threatening infections and portal vein thrombosis that can arise from post-splenectomy thrombocytosis and hypercoagulability. Thus, early diagnosis holds significant value (12).

Given the severe torsion and partial splenic infarction observed, splenectomy was deemed the most appropriate surgical intervention. Intraoperatively, a 360° torsion of the splenic pedicle and areas of partial necrosis were found, rendering the spleen non-viable. This case underscores the critical need for prompt surgical intervention in acute splenic torsion and highlights the importance of a multidisciplinary approach to optimize patient outcomes.

Postoperatively, the patient received subcutaneous injections of low molecular weight heparin sodium, followed by oral aspirin therapy. This individualized approach, combined with regular monitoring, effectively managed the patient’s condition for patients following splenectomy. The spleen, the largest lymphoid organ, is crucial for blood filtration. Following splenectomy, patients typically experience a significant rise in red blood cell and platelet counts, which can lead to hypercoagulability and an increased risk of venous thrombosis. Antiplatelet therapy is essential for postoperative anticoagulation (13). Given the high risk of venous thrombosis associated with splenectomy, appropriate anticoagulation is critical. There is currently no consensus on the optimal anticoagulation regimen following splenectomy (14). Generally, anticoagulant drugs are administered early after surgery, barring any significant bleeding risk (15). Some studies suggest initiating anticoagulation when the platelet count exceeds 500 × 10^9^/L, with options including low molecular weight heparin sodium, aspirin, clopidogrel, dipyridamole, rivaroxaban, and warfarin. Anticoagulation can be discontinued once the platelet count normalizes. Regular monitoring of platelet counts, coagulation parameters, and ultrasound examinations are vital for detecting thrombosis. The treatment plan should be individualized based on the patient’s condition (16).

Splenectomy significantly increases the risk of overwhelming postsplenectomy infection (OPSI). To mitigate these risks, vaccination against encapsulated organisms (pneumococcus, meningococcus, and *Haemophilus influenzae* type B) is strongly recommended. Prophylactic antibiotics, particularly in the first few years post-splenectomy, further reduce the risk of severe infections. These preventive measures are crucial for long-term management and improving patient outcomes.

## 4 Conclusion

Wandering spleen is a rare clinical condition with a high rate of misdiagnosis and potentially serious complications following splenic torsion. Early diagnosis and appropriate treatment are essential. For patients who require splenectomy due to severe torsion, timely surgical intervention and postoperative anticoagulant therapy are critical for saving lives and preventing complications. This case highlights the importance of maintaining a high index of suspicion, utilizing early diagnostic imaging, and adopting a multidisciplinary approach in managing wandering spleen. The detailed management of this case offers valuable insights for clinicians dealing with similar rare and challenging conditions. Increased awareness among primary care providers and emergency teams is crucial to facilitate timely recognition and intervention in such cases. Importantly, our patient remained asymptomatic at 2-month follow-up with no evidence of thrombosis, demonstrating the effectiveness of the management strategy employed.

## Data Availability

The original contributions presented in the study are included in the article/supplementary material, further inquiries can be directed to the corresponding author.
